# Mouth Breathing; Its Cause and Its Consequences

**Published:** 1908-05-15

**Authors:** Woods Hutchinson


### MOUTH BREATHING; ITS CAUSE AND ITS CONSEQUENCES.

BY WOODS HUTCHINSON, A M., MD.

In all ages it has been accounted a virtue to keep your mouth shut—chiefly, of course, upon moral or prudential grounds, for fear of what might issue from it if opened. Then came physiology to back up the maxim, on the ground that the open mouth was also dangerous on account of what might be inhaled into it. Oddly enough, in this instance, both morality and science have been beside the mark to the degree that they have been mistaking a symptom for a cause. This has led us to absurd and injurious extremes in both cases. On the moral and prudential side it has led to such outrageous exaggerations as the well-known and oft-quoted proverb: "Speech is silver, but silence is golden." Articulate speech, the chiefest triumph and highest single accomplishment of the human species, the handmaid of thought and the instrument of progress, is actually rated below silence, the attribute of the clod and of the dumb brute, the easy refuge of cowardice and of stupidity.

Easily eight-tenths of all speech is informing, educative, helpful in some modest degree, while fully that proportion of silence is due to lack of ideas, cowardice, or designs that can flourish only in darkness. It is not the abundance of words, but the scarcity of ideas, that makes us flee from "the plugless word-spout" and avoid the chatterbox.

Similarly, upon the physical side, because children who breathe through the mouth are apt to have a vacant expression, to be stupid and inattentive, undersized, pigeonbreasted, with short upper lip and crowded teeth, we have leaped to the conclusion that it is a fearsome and dangerous thing to breathe through your mouth. All sorts of stories are told about the dangerousness of breathing frosty air directly into the lungs. Invalids shut themselves scrupulously indoors for weeks and even months at a stretch, for fear of the terrible results of a "blast of raw air" striking into their bronchial tubes. All sorts of absurd instruments of torture in the form of "respirators" to tie over the mouth and nose and "keep out the fog" are invented, and those who have the slightest tendency to bronchial or lung disturbances are warned upon pain of their life to wrap up their mouths whenever they go out-of-doors.

As a matter of fact, there is exceedingly little evidence to show that pure, fresh, open air at any reasonable temperature and humidity ever did harm when inhaled directly into the lungs. In fact, a considerable proportion of us, when swinging along at a lively gait on the country roads, or playing tennis or foot ball, or engaged in any form of active sport, will be found to keep our lips parted and to inhale from a sixth to a third of our breath in this way, and with no injurious results whatever. Nine-tenths of all the maladies believed to be due to breathing even the coldest and rawest of air are now known to be due to invading germs.

Nevertheless, mouth breathing in all ages has been regarded as a bad habit, and with good reason. It was only about thirty years ago that we began to find out why. A Danish throat surgeon, William Meyer, whose death occurred only a few months ago, discovered, in studying a number of children who were affected with mouth breathing, that in all of them there were present in the roof of the throat curious spongy growths which blocked up the posterior opening of the nostrils. As this mass was made up of a number of smaller lobules, and the tissue appeared to be like that of a lymphatic gland, or "kernel," the name "*adenoids*" ("glandlike") was given to them. Later they were termed *post-nasal growths*, from the fact that they lay just behind the rear opening of the nostrils, and these two names are used interchangeably. Our knowledge has spread and broadened from this starting point, until we now know that adenoids are the chief, yes, almost the sole cause, not merely of mouth breathing, but of at least two-thirds of the injurious effects which have been attributed to this habit.

Mouth breathing is not simply a bad habit, a careless trick on the part of the child. We have come to realize that physical bad habits, as well as many mental and moral ones, have a definite physical cause, and that *no child ever becomes a mouth breather as long as he can breathe comfortably through his nose.*

This clears the ground at once of a considerable amount of useless lumber in the shape of advice to train the child to keep his mouth shut. I have even known mothers who were in the habit of going around after their helpless offspring were asleep and gently but firmly pushing up the little jaw and pressing the lips together until some sort of an attempt at respiration was made through the nostrils. Advertisements still appear of slinglike apparatuses for holding the jaws closed during sleep.

To attempt to stop mouth breathing before providing abundant air-space through the nostrils is not only irrational, but cruel. Of course, after the child has once become a mouth-breather, even after the nostrils have been made perfectly free, it will not at once abandon its habit of months or years, and disciplinary measures of some sort may the be needed for a time. But the hundred-times-repeated admonition; "For Heaven's sake, child, shut your mouth! Don't go around with it hanging open like that!" unless preceded by proper treatment of the nostrils, will have just about as much effect upon the habit as the proverbial water on a duck's back. No use trying to close his mouth by any amount of opening of your own.

Fortunately, as does not always happen, with our discovery of the cause has come the knowledge of the cure, and we are able to say with confidence that, widespread and serious as are disturbances of health and growth associated with mouth breathing, they can be absolutely prevented and abolished.

What, then, is the cause of this nasal obstruction, and when does it begin to operate? The primary cause is catarrhal inflammation, with swelling and thickening of the secretions, and it may begin to operate anywhere from the seventh month to the seventh year. A neglected attack or series of attacks of "snuffles," 'colds in the head," "catarrhs" in infants and young children, will set up a slow inflammation of this glandular mass at the back of the nostrils—a tonsil, by the way—and start its enlargement.

Whether we know anything about adenoids themselves or not, we are all familiar with their handiwork. The open, mouth, giving a vacant expression to the countenance, the short upper lip, the pinched and contracted nostrils, the prominent and irregular teeth, the listless expression of the eyes, the slow response to request or command, we have seen a score of times in every schoolroom. Coupled with these facial features are apt to be found on closer investigation a lack of interest in both work and play, an impaired appetite, restless sleep and a curious general backwardness of development, both bodily and mental, so that the child may be from one to four inches below the normal height for his years, from five to fifteen pounds under weight and from one to three grades behind his proper school position. Very often, also, his chest is inclined to be narrow, the tip of his breast-bone to be sunken in, and his abdomen larger in girth than his chest. Is it possible that the mere inhaling of air directly into the lungs, even though it be imperfectly warmed, moistened and filtered, as compared with what it would be if drawn through the elaborate "steam-coils" in the nostrils for this purpose, can have produced this array of defects? It is incredible on the face of it and unfounded in fact. Fully two-thirds of these can be traced to the direct influence of the adenoids.

These adenoids, it may be briefly stated, are the result of an enlargement of a *tonsil*, or group of small tonsils, identical in structure with the well-known bodies of the same name which can be seen on either side of the throat. They have the same unfortunate faculty as the other tonsils for getting into hot water, flaring up, inflaming and swelling on the slightest irritation. And, unfortunately, they are so situated that their capacity for harm is far greater than that of the other tonsils. They seem painfully like the chip on the shoulder of a fighting man, ready to be knocked off at the lightest touch and plunge the whole body into a scrimmage. Their position is a little difficult to describe to one not familiar with the anatomy of the throat, especially as they cannot be seen except with a laryngeal mirror. But it may be roughly stated as in the middle of the roof of the throat, just at the back of the nostrils, and above the soft palate. From this coign of vantage they are in position to produce serious disturbances of two of our most important functions—respiration and digestion, and three of the five senses—smell, taste and hearing.

We will begin with their most frequent and most serious injurious effect, though not the earliest—the impairment of the child's power of attention and intelligence. So well known is their process in this respect that there is scarcely an intelligent and progressive teacher nowadays who is not thoroughly posted on adenoids. Some of them will make a snap diagnosis as promptly and almost as accurately as a physician: and when once they suspect their presence they will leave no stone unturned to secure an examination of the child by a competent physician, and the removal of the growths, if present. They consider it a waste of time to endeavor to teach a child weighted with this handicap. How keenly awake they are to their importance is typified by the remark of a prominent educator five or six years ago;

"When I hear a teacher say that a child is stupid my first instinctive conclusion is either that the child has adenoids, or that the teacher is incompetent."

The lion's share of their influence upon the child's intelligence is brought about in a somewhat unexpected and even surprising manner, and that is by the *effects of the growths upon his hearing.* You will recall that this third tonsil was situated at the highest point in the roof of the pharynx, or back of the throat. The first effect of its enlargement is naturally to block the posterior opening of the nostrils. But it has another most serious vantage-ground for harm in its peculiar position. Only about three-fourths of an inch below it upon either side open the mouths of the Eustachian tubes, the little funnels which carry air from the throat out in to the drum cavity of the ear. You have frequently had practical demonstrations of their existence, by the well-known sensation, when blowing your nose vigorously, of feeling something go "pop" in the ear. This sensation was simply due to a bubble of air being driven out through this tube from the back of the throat under pressure brought to bear in blowing the nose. The luckless position of the third tonsil could hardly have been better planned if it had been devised for the special purpose of setting up trouble in the mouths of these Eustachian tubes.

Just as soon as the enlargements become chronic they pour out a thick mucous secretion, which quickly becomes purulent, or, in the vernacular, "matter." This trickles down on both sides of the throat, and drains right into the open mouth of the Eustachian tube. Not only so, but these Eustachian tubes are the remains of the first gill-slits of embryonic life, and, like all other gill-slits, have a little mass of this same lymphoid or tonsilar tissue surrounding them. This also becomes infected and inflamed, clogs the opening, and one fatal day the inflammation shoots out along the tube, and the child develops an attack of earache. At least two-thirds of all cases of earache, and, indeed, five-sixths of all cases of deafness in children, are due to adenoids.

Earache is simply the pain due to acute inflammation in the small drum cavity of the ear. This in the large majority of cases will subside and drain back again into the throat through the Eustachian tube. In a fair percentage of instances, however, it will break in the opposite direction, and we have the familiar ruptured drum and discharge from the ear. In either case the drum becomes thickened, so that it can no longer vibrate properly, the delicate little chain of bones behind it, like the levers of the piano, becomes clogged, and the child becomes deaf, whether a chronic discharge be present are not.

This is the secret of his inattention, his "indifference" —even of his apparent disobedience and rebelliousness. What other children hear without an effort he has to strain every nerve to catch. He misunderstands the question that is asked of him, makes an absurd answer, and is either scolded or laughed at. It isn't long before he falls into the attitude; "Well, I can't get it right anyhow, no matter how I try, so I don't care." Up to five or ten years ago the puzzled and distracted teacher would simply report the child for stupidity, indifference and even insubordination. In nine cases out of ten when children are naughty or stupid they are really sick.

Not content with dulling one of the child's senses, these thugs of the body politic proceed to throttle two others—smell and taste. Obviously the only way of smelling anything is to sniff its odor into your nose. And if this be more or less, or completely, blocked up, and its delicate mucous membranes coated with a thick, ropy discharge, you will not be able to distinguish anything but the crudest and rankest of odors. But what has this to do with taste? Merely that two-thirds of what we term "taste" is really smell. Seal the nostrils and you can't tell chalk from cheese,"' not even a cube of apple from a cube of onion, as scores of experiments have shown. We all know how flat tea, coffee and even our favorite dishes taste when we have a bad cold, and this, remember, is the permanent condition of the palate of the poor little mouth-breather. No wonder his appetite is apt to be poor, and that even what food he eats will not produce a flow of "appetite juice" in the stomach, which Pavloff has shown to be so necessary to digestion. No wonder his digestion is apt to go wrong, ably assisted by the continual drip of the chronic discharge down the back of his throat, his bowels to become clogged and his abdomen distended.

But the resources for mischief of this pharyngeal "Old Man of the Sea" are not even yet exhausted. Next comes a very curious and unexpected one. We have all heard much of the "the struggle for existence" among plants and animals and had painful demonstrations of its realty in our own personal experience. But we hardly suspected that it was going on in our own interior. Such, however, is the case, and when once one organ or structure falls behind the others in the race of growth, its neighbors promptly begin to encroach upon and take advantage of it. Emerson was right when he said, "I am the Cosmos," the universe.

Now, the mouth and the nose were originally one cavity. As Huxley long ago remarked; "When Nature undertook to build the skull of a land animal she was too lazy to start on new lines, and simply took the old fish skull and made it over, for air-breathing purposes." And a clumsy job she made of it! It may be remarked, in passing, that mouth breathing, as a matter of history, is an exceedingly old and respectable habit, a reversion, in fact, to the method off breathing of the fish and the frog.

"To drink like a fish" is a shameful and utterly unfounded aspersion upon a blameless animal of most correct habits and model deportment. What the poor gold fish in the bowl is really doing with his continual "gulp, gulp!" is breathing—not drinking.

This remodeling begins at a very early period of our individual existence. A horizontal ridge begins to grow out on either side of our mouth-nose cavity, just above the roots of the teeth. This thickens and widens into a pair of shelves, which finally, about the third month of embryonic life, meet in the middle line to form the hard palate or roof of the mouth, which forms also the floor of the nose. Failure of the two shelves to meet properly causes the well-known "*cleft-palate,*" and if this failure extends forward to the jaw, *hare-lip*. In the growth of a healthy child a balance is preserved between these lower and upper compartments of the original mouth-nose cavity, and the nose above growing as rapidly in depth and in breadth, as the mouth below, and the horizontal partition between, the floor of the nose and the roof of the mouth is kept comparatively flat and level. In adenoids, however, the nostrils no longer being adequately used, and consequently failing to grow, and the mouth cavity below growing at the full normal rate, it is not long before the mouth begins to encroach upon the nostrils by pushing up the partition of the palate. As soon as this upward bulge of the roof of the mouth occurs, then there is a diminution of the resistance offered by the horizontal healthy palate to the continual pressure of the muscles of the cheeks and of mastication upon the sides of the upper jaw—the more readily as the tongue has dropped down from its proper resting position up in the roof of the mouth. These are pushed inward, the arch of the jaw and of the teeth is narrowed, the front teeth are made to project, and, instead of erupting, with plenty of room, in even, regular lines, are crowded against and overlap one another.

In short, we are coming to the conclusion that from half to two-thirds of all cases of "crowded mouth," irregular teeth and high-arched palate in children are due to adenoids.

Progressive dentists now are insisting upon their little patients who come to them with these conditions, being examined for adenoids, and the removal of these, if found, as a preliminary measure to any mechanical corrective treatment. Cases are now on record of children with two, three or even four generations of crowded teeth and narrow mouths behind them, but who, simply by being sharply watched for nasal obstruction and the symptoms of adenoids, and these latter removed as soon as they have put in an appearance, have grown up with even, regular, well-developed teeth and wide, healthy mouths and jaws. Unfortunately, attention to the adenoids will not by any means always remove these defects of the jaws and teeth after they have been produced. But, if the child be under ten, or even twelve, years of age, their removal may yet do much to permanently improve the condition, and is certainly well worth while on general principles.

Take care of the nose, and the jaws will take care of themselves. An ounce of adenoids-removal in the young child is worth a pound of *orthodontia*—teeth-straightening—in the boy or girl.

The dull, dead tone of the voice in these children is, of course, an obvious effect of the blocked nostrils. Similarly the broken sleep, with dreams of suffocation and of "Things Sitting on the Chest," are readily explained by the desperate efforts that the little one makes to breathe through clogging nostrils, in which the discharges, blown and sneezed out in the daytime, dry and accumulate during sleep, until, half suffocated, it "lets go" and draws in huge gulps of air through the open mouth. No child ever became a mouth breather from choice, or until after a prolonged struggle to continue breathing through its nose.

This brings us to the question, What are these adenoids, and how do they come to produce such serious disturbances? This can be partially answered by saying that they are tonsils and with all a tonsil's susceptibility to irritation and inflammation. But that only raises the further question, What is a tonsil? And to that no answer can be given but Echo's. They are one of the conundrums of physiology. All we know of them is that they are not true *glands*, as they have neither duct nor secretion, but masses of simple embryonic tissue called *lymphoid*, which has a habit of grouping itself about the openings of disused canals. This is what accounts for their position in the throat, as they have no known useful function. The two largest, or throat tonsils, surround the inner openings of the second gill-slits of the embryo; the lingual tonsil, at the base of the tongue below, encircles the mouth of the duct of the thyroid gland (the "*goitre*" gland); and our own particular Pandora's Box above, in the roof of the pharynx, is grouped about the opening of another disused canal, which performs the singular and apparently most uncalled-for office of connecting the cavity of the brain with the throat. They can all of them be removed completely without any injury to the general health, and they all tend to shrink, become smaller—in the case of the topmost or pharyngeal, almost disappear—after the twelfth or fourteenth year.

Not only have they an abundant crop of troubles of their own, as most of us can testify from painful experience, but they serve as a port of entry for the germs of many serious diseases, such as tuberculosis, rheumatism, diphtheria and possibly scarlet fever. They appear to be a strange sort of survival or remnant—not even suitable for the bargain-counter—a hereditary leisure class in the modern democrary of the body, a fertile soil for all sorts of trouble.

Here, then, we have this little bunch of idle tissue, about the size of a small hazelnut, ready for any mischief which our Satan-bacilli may find for its hands to do. A child kept in a badly ventilated room inhales into its nostrils irritating dust or gases, or more commonly yet, the floating germs of some one or more of those dozen mild infections which we term "a common cold." Instantly irritation and swelling are set up in the exquisitely elastic tissues of the nostrils, thick, sticky mucus, instead of the normal, watery secretion, is poured out, the child begins to sneeze and snuffle and "run at the nose," and either the bacteria are carried directly to this danger sponge, right at the back of the nostrils, or the inflammation gradually spreads to it. The mucous membrane and tissues of the nose have an abundance of vitality—like most hard workers—and usually react, overwhelm and destroy the invading germs and recover from the attack; but the useless and half-dead tissue of the pharyngeal tonsil has much less power of recuperation, and it smoulders and inflames, though ultimately, perhaps, it may swing round to recovery. Often, however, a new cold will be caught before this has fully occurred, and then another one a month or so later, until we finally get a chronically thickened, inflamed and enlarged condition of this interesting, but troublesome, body. What its capabilities are in this respect may be gathered from the fact that, while normally of the size of a small hazelnut, it is no uncommon thing to find a mass which absolutely blocks up the whole of the upper part of the pharynx, and may vary from the size of a robin's egg to that of a large English walnut, or even a small hen's egg, according to the age of the child and the size of the throat.

Dirt has been defined as "matter out of place," and the pharyngeal tonsil is an excellent illustration. Nature is said never to make mistakes, but she is apt to be absent-minded at times, and we are tracing now not a few of the troubles that our flesh is heir to—to little oversights of hers—scraps of inflammable material left lying about among the cogs of the body-machine, such as the appendix, the gall-bladder, the wisdom teeth and the tonsils. One day a spark drops on them, or they get too near a bearing or a "hot-box," and, with a flash, the whole machine is in a blaze.

Never neglect snuffles or "cold in the head" in a young child, and particularly in a baby. Have it treated at once antiseptically by competent hands, and learn exactly what to do for it on the appearance of the earliest symptoms in the future, and you will not only save the little ones a great deal of temporary discomfort and distress—for it is perfect torment for a child to breath through its mouth at first—but you will ward off many of the most serious troubles of infancy and childhood. We can hardly expect to prevent all development of adenoids by these prompt and painless stitches in time, for some children seem to be born peculiarly subject to them, either from the inheritance of a particular shape of nose and throat—"the family nose," as it has been called,—or from some peculiar sponginess and liability to inflammation and enlargement of all these tonsillar or lymphoid "glands" and "kernels" of the body generally—the old "lymphatic temperament."

We are, however, now coming to the opinion that this so-called "hereditary" narrow nose, short upper lip and higharched palate are, in a large percentage of cases, the *result of adenoids in infancy* in each successive generation of parents and grandparents. At all events, there are now on record cases of children whose parents, grand-parents and great-grandparents are known to have been mouth-breathers, and who have on that account been sharply watched for the possible development of adenoids in early life, and these removed as soon as they appear, and they have grown up with well-developed, wide nostrils, broad, flat palates and regular teeth overcoming "hereditary defect" in a single generation.

Curiously enough, their origin and ancestral relations may have an important practical bearing, even in the twentieth century. At the upper end of this curious *throat-brain* canal lies another mass, the so-called *pituitary body*. This has been found to exert a profound influence over development and growth. Its enlargement is attended by giantism and another curious giant disease in which the hands, feet and jaws enlarge enormously, known as *acromegaly*. It also pours into the blood a secretion which has a powerful effect upon both the circulation and the respiration. It is found shrunken and wasted in dwarfs. Some years ago it was suggested by my distinguished friend, the late Dr. Harrison

Allen, and myself that some of the extraordinary dwarfing and growth-retarding effects of adinoids might be due to a reflex influence exerted on their old colleague, the pituitary body. This view has found its way into several of the textbooks.

Blood is thicker than water, and old ancestral vibrations will sometimes be set up in most unexpected places.

Now comes the cheerful side of the picture. I should have hesitated to draw at such full length and in such lugubrious detail the direful possibilities and injurious effects of adenoids if its only result could have been to arouse apprehensions which could not be relieved.

Fortunately, just the reverse is the case, and there are few conditions affecting the child so common and such a fertile source of all kinds of mischief, and at the same time so completely curable, and whose cure will be attended by such gratifying improvement on the part of the little sufferer. In the first place, as has been said, their formation may usually be prevented altogether by intelligent and up-to-date hygienic care of the nose and the throat. In the second place, even after they have occurred and developed to a considerable degree they can be removed by a trifling and almost painless operation, and, if taken early enough, all their injurious effects overcome. If, however, they have been neglected too long, so that the child has passed the eighth or ninth year before any interference has been attempted, and still more, of course, if it has passed the twelfth or thirteenth year, then only a part of the disturbances that have been caused can be remedied by their removal. So soft and pulpy are these growths, so poorly supplied with blood-vessels or nerves, and so slightly connected with the healthy tissues below them, that they may, in skilled hands, be completely removed by simply scraping with a dull surgical spoon (curette) or curved forceps, but never anything more knifelike than this. In fact, in the first seven years of life, when their removal is both easiest and will do most good, it is hardly proper to dignify the procedure by the name of an operation.

It is attended by about the same degree of risk and of hemorrhage as the extraction of a tooth, and by less than half the amount of pain.

But trifling and free from danger as is the operation, there is nothing in the entire realm of surgery which is followed by more brilliant and gratifying results. It seems almost incredible until one has seen it in half a dozen successive cases. Not merely doctors, but teachers and nurses, develop a positive enthusiasm for it. This was the operation that led to the comical, but pathetic, "Mothers' Riots" in the New York schools. The word went forth; "The Krishts are cutting the throats of your children," and with shameful echoes of Kishineff ringing in their ears, the Yiddish mothers swarmed forth to battle for the lives of their offspring. It is no uncommon thing to have a child of seven jump three to five inches in height, six to twelve pounds in weight and one to three grades in his schooling within the year following the operation. Ten years more of intelligence and hygienic teaching should see this scourge of childhood completely wiped out, or at least robbed of its possibilities for harm. When this is done at least two-thirds of all cases of deafness, more than half of all cases of arrested development and three-fourths of backwardness in children will disappear.—*Saturday Evening Post,* April 4th, 1908.

